# Improving the enzymatic activity and stability of N-carbamoyl hydrolase using deep learning approach

**DOI:** 10.1186/s12934-024-02439-5

**Published:** 2024-06-04

**Authors:** Fa Zhang, Muhammad Naeem, Bo Yu, Feixia Liu, Jiansong Ju

**Affiliations:** 1 College of Life Science, Hebei Normal University, Shijiazhuang, 050024 China; 2 Institute of Microbiology, Chinese Academy of Sciences, Beijing, 100101 China; 3 Hebei Collaborative Innovation Center for Eco-Environment, Shijiazhuang, 050024 China

**Keywords:** N-carbamoyl-D-amino acid amidohydrolase, Molecular dynamics simulation, Rational design, Deep Learning module, D-p-hydroxyphenylglycine

## Abstract

**Background:**

Optically active D-amino acids are widely used as intermediates in the synthesis of antibiotics, insecticides, and peptide hormones. Currently, the two-enzyme cascade reaction is the most efficient way to produce D-amino acids using enzymes DHdt and DCase, but DCase is susceptible to heat inactivation. Here, to enhance the enzymatic activity and thermal stability of DCase, a rational design software “Feitian” was developed based on *k*_cat_ prediction using the deep learning approach.

**Results:**

According to empirical design and prediction of “Feitian” software, six single-point mutants with high *k*_cat_ value were selected and successfully constructed by site-directed mutagenesis. Out of six, three mutants (Q4C, T212S, and A302C) showed higher enzymatic activity than the wild-type. Furthermore, the combined triple-point mutant DCase-M3 (Q4C/T212S/A302C) exhibited a 4.25-fold increase in activity (29.77 ± 4.52 U) and a 2.25-fold increase in thermal stability as compared to the wild-type, respectively. Through the whole-cell reaction, the high titer of D-HPG (2.57 ± 0.43 mM) was produced by the mutant Q4C/T212S/A302C, which was about 2.04-fold of the wild-type. Molecular dynamics simulation results showed that DCase-M3 significantly enhances the rigidity of the catalytic site and thus increases the activity of DCase-M3.

**Conclusions:**

In this study, an efficient rational design software “Feitian” was successfully developed with a prediction accuracy of about 50% in enzymatic activity. A triple-point mutant DCase-M3 (Q4C/T212S/A302C) with enhanced enzymatic activity and thermostability was successfully obtained, which could be applied to the development of a fully enzymatic process for the industrial production of D-HPG.

**Supplementary Information:**

The online version contains supplementary material available at 10.1186/s12934-024-02439-5.

## Background

Optically active D-amino acids are widely used as intermediates for the synthesis of antibiotics, insecticides, and peptide hormones. Specifically, D-p-hydroxyphenylglycine (D-HPG) is one of the important intermediates in the industrial production of antibiotics such as amoxicillin, penicillin, and cephalosporins [1–3]. The annual market requirements of D-HPG is about 10,000 tons. Therefore, an efficient synthesis process of D-HPG is urgently required to meet the high demand of society [4].

Currently, chemical and biocatalytic methods are commonly used for the production of D-HPG [5]. In both methods, the biocatalytic asymmetric synthesis of D-HPG has gained much attention due to mild conditions and low pollution [6, 7]. There are two main biocatalytic methods for the production of D-HPG: the four-enzyme cascade method and the two-enzyme cascade method [8–10]. The two-enzyme catalysis method exhibits a much higher conversion rate than the four-enzyme cascade method. However, the space–time yield (STY) through this method is relatively low due to the low stability and product inhibition of N-carbamoyl-D-amino acid amidohydrolase (DCase, EC 3.5.1.77) [10]. The enzyme DCase facilitates the hydrolysis of the N-carbamoyl group in the production of N-carbamoyl-D-amino acids. It is employed in the industrial production of *D, L*-5-monosubstituted barbiturates, which catalyzes the opening of the D-hydantoin ring and produces the N-carbamoyl-D-amino acids alongside D-hydantoinase (DHdt, EC. 3. 5. 2. 2) [11]. The low activity of enzyme DCase might be attributed to the hydroxyl occupying effect [9].

Different methods for DCase enzyme engineering were reported for achieving maximum thermostability. Ikenaka et al. applied a PCR random mutagenesis strategy for introducing the mutations into the DCase enzyme of *Agrobacterium* sp. KNK712 strain. Among the generated mutants, the mutant H57Y/P203E/V236A exhibited a remarkable increase of 10 ℃ in thermal stability [12]. Oh et al. conducted directed evolution through DNA recombination to introduce mutations into the DCase enzyme of *Agrobacterium tumefaciens* strain NRRL B11291, and the mutant Q23L/V40A/H58Y/G75S/M184L/T262A showed thermal stability up to 73 ℃ [13]. Chiu et al. reported that the disulfide bonds were introduced into DCase (A302C) of *A. radiobacter* resulting in a 4.2-fold increase in *k*_cat_/*K*_*m*_ value at 65 °C [14]. Jiang et al. successfully generated a three-point mutant (A18T/Y30N/K34E) using error-prone PCR and DNA shuffling techniques, which showed a three-fold increase in solubility as compared to wild-type [15]. Based on this result, a stepwise evolution method was employed to enhance the thermal stability of the mutant. After screening, one thermal stability with 10-degree improvement was attained as compared to the mutant A18T/Y30N/K34E [16]. Finally, the thermal stability of AkDCase was improved through salt bridge engineering. The optimized variant, AkDCaseD30A, showed an 2.91 °C increase in the melting temperature (Tm) [10]. Overall, these methods are time-consuming, cumbersome, and expensive. Therefore, an alternative rational design is needed to enhance the enzymatic activity and thermal stability of DCase.

In this study, the “Feitian” software was developed for rational design for DCase using a deep learning model. Through this software, six single-point mutants with high *k*_cat_ value were predicted, then 25 single, 3 double, and 4 triple-point mutants were constructed by site-directed mutagenesis and site-saturation mutagenesis. Protein expression, purification, enzymatic characteristics, and structural modeling of DCase and its mutants were carried out. Furthermore, the whole-cell reaction for D-HPG production was also investigated.

## Materials and methods

### Strains, plasmids, and media

The plasmids pET-28a(+), pYB1s*,* and host strains *E. coli* BL21(DE3) & MG1655(DE3) were used for protein expression and CpHPG production, respectively. Luria Bertani (LB) liquid or solid media was utilized for inoculum cultivation, kanamycin (50 μg/mL) and IPTG (0.4 mM) was added to medium when necessary. 5-(4-hydroxyphenyl) hydantoin and 2-amino-2-(4-hydroxyphenyl) acetic acid were purchased from Shanghai Gezone Bioscience Co., Ltd. All other chemicals and reagents used were of analytical grade and purchased from commercial sources.

HPG was synthesized from DL-HPH via the cascade of DHdt (EC 3.5.2.2) and DCase (EC 3.5.1.77) [17]. The Hase used in this study was a double-point mutation (M63I/F159S) of carbamoylase with increased activity towards DL-HPH [18]. The *hase* and *dcase* genes were inserted into pYB1s and pET-28a(+) using Gbison assembly method for the construction of plasmids pYB1s-Hase and pET-28a(+)-Case, respectively. The plasmid pYB1s-Hase was transformed into strain MG1655(DE3) for production of intermediate product CpHPG. pET-28a(+)-Case was then transformed into BL21(DE3) for protein expression and purification.

### Preparation of the intermediate N-carbamoyl-D-p-hydroxyphenylglycine

Substrate CpHPG for the DCase-catalyzed reaction was initially prepared due to non-commercialization of the reaction intermediate N-carbamoyl-D-p-hydroxyphenylglycine (CpHPG). Strain MG1655 (DE3) harboring plasmid pYB1s-Hase was induced by the addition of 0.2% (v/v) arabinose at 37 ℃ for 12 h, and the induced cells were centrifuged and resuspended in reaction mixture with OD_600_ value around 30. The transformation reaction mixture was composed of 50 mM Tris–HCl buffer (pH 7.5) containing 20 mM DL-hydroxyphenylhydantoin (DL-HPH), 1.0 mM MnCl_2_, and 1.0 mM 1,4-Dithiothreitol (DTT), and carried out at 50 ℃ with shaking at 200 rpm for 12 h. After the removal of bacterial cells by centrifugation, the supernatant containing CpHPG was stored at 4 ℃ and used as the substrate for DCase characterization.

### Screening for targeted mutation sites of DCase

**Fig. 1:**
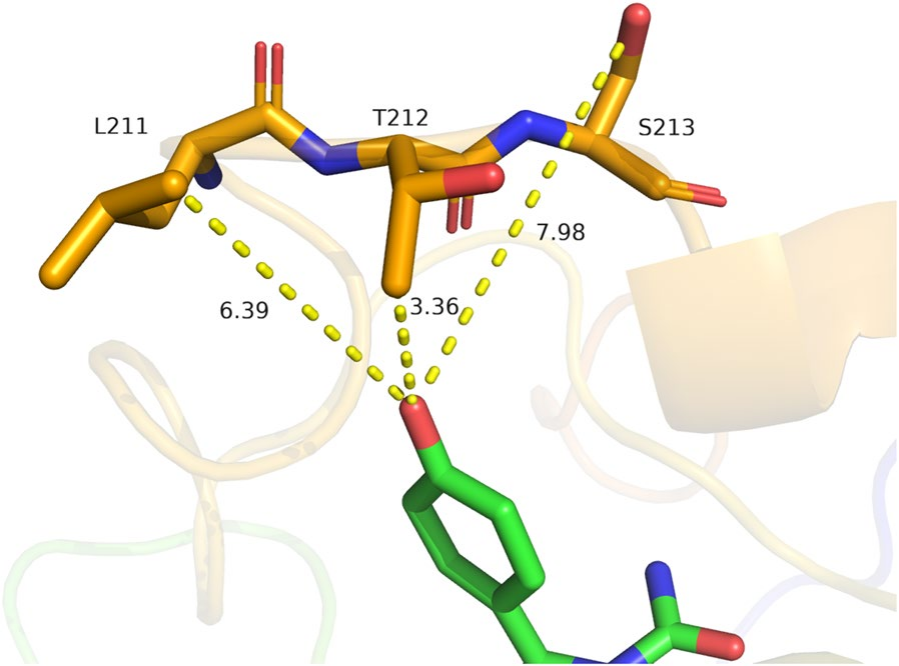
Interaction of phenyl hydroxyl groups of small molecules (CpHPG) with DCase

Two methods were employed for virtual screening to predict mutations with improved activity towards CpHPG. Firstly, residues at N-terminal and C-terminal positions 3, 4, 302, and 303 were selected. The N-terminal and C-terminal readily react with other compounds through different reactions, such as acylation and esterification. The amino acid residues and their side chain of N&C-terminal could also affect the folding and stability of proteins. Therefore, 4 residues (3, 4, 302, 303) were selected close to each other in the loop, β-folding and α-helix at N-termini and C-termini. Secondly, the molecular docking results revealed that residue 212 was the nearest site from the phenyl hydroxyl of molecule CpHPG (3.36 Å), which resulted in a decrease in substrate affinity due to the steric hindrance of the side chain of CpHPG (Fig. 1).

The *k*_cat_ values of all sites were predicted using the software “Feitian” and partially shown in Table S1. The* k*_cat_ value of 5 sites (3, 4, 212, 302, and 303) were listed in Table S2 and further analyzed by screening method [19]. According to the following criteria, these mutations were filtered out from experimental validations: (1) mutation may severely disrupt salt-bridge interactions or hydrogen bonds (R3I, Q4T, T212Y, T212M, E303H, and E303R); (2) mutation may result in steric clashes with the remaining structures (Q4N, T212P, T212C, and E303N); (3) residues substituted by hydrophobic residues located on the protein surface (Q4R, Q4D, T212S and E303K). After exclusion, 6 mutations with the highest *k*_cat_ values at each locus were selected (R3E, *k*_cat_ 23.08; Q4C, *k*_cat_ 21.11; T212S, *k*_cat_ 19.90; A302Y, *k*_cat_ 19.89; A302C, *k*_cat_ 19.89; E303Q, *k*_cat_ 18.19), and site-directed mutagenesis was performed by PCR using high-fidelity Q5 DNA polymerase using plasmid pET-28a(+)-Case as the template. The nucleotide sequences of primers used for mutagenesis are shown in Table S3. PCR products were digested by the restriction enzyme *Dpn*I at 37 °C for 3 h. All constructed mutants were verified by DNA sequencing.

### Protein expression and purification

All plasmids were transformed into *E. coli* BL21 (DE3) and incubated on LB agar medium overnight at 37 ℃. A single positive colony was picked and inoculated into LB medium containing 50 μg/mL of kanamycin and cultured at 37 ℃ and 200 rpm. When cell density at 600 nm (OD_600_) approached near 0.6, the cells were induced by with 0.4 mM addition of isopropyl thio-*β*-D-galactoside (IPTG) for 16 h at 18 °C. The expressed cells were harvested by centrifugation at 8000 rpm and 4 °C for 15 min, and harvested cells were resuspended in binding buffer (20 mM Tris–HCl, pH 7.4, 20 mM imidazole, 500 mM NaCl). After ultrasonication and centrifugation, the recombinant protein with His_6_-tag in supernatant was purified by nickel affinity chromatography. The eluted protein was then desalted, concentrated, and dialyzed against 20 mM Tris–HCl (pH 7.5) by ultrafiltration with an Amicon Ultra-15 centrifugal filter device (30 K MWCO, Millipore). The purity of the protein was evaluated by sodium dodecyl sulfate polyacrylamide gel electrophoresis (12% SDS-PAGE). Protein concentration was quantified by the bicinchoninic acid (BCA) method (Pierce, USA).

### Enzymatic assay

The activity of DCase and all variants towards CpHPG was analyzed by HPLC with a Poroshell 120 EC18 column (2.7 μm, 4.6 mm × 50 mm; Agilent, California, USA). The reaction was carried out at 65 ℃ for 20 min in a reaction system containing 10 mM CpHPG and 0.10–0.60 mg/mL enzyme, and it was terminated at 100 °C for 3 min. The amounts of D-HPG were determined by HPLC analysis with a mobile phase consisting of 0.1% (v/v) formic acid solution (90%) and acetonitrile (10%) at a flow rate of 0.5 mL/min. One unit (U) of enzyme is defined as the amount of DCase that generates 1 mmol of D-HPG within 1 min.

### Thermostability assays

The purified proteins were incubated at 60 °C for 20 min in Tris–HCl buffer (20 mM, pH 7.5). Then, 10 µL of heat-treated protein (5.0 mg/mL) was added into 90 µL of the reaction solution containing 5 mM CpHPG and kept at 60 °C for 5 min. The reaction mixtures was then boiled for 3 min and stopped the reaction. The thermostability of wild-type and variants was evalutated by measuring the residual activity using the HPLC method.

### Kinetic assays

The kinetic parameters of the wild-type and mutants were assessed and the amount of D-HPG was determined by HPLC analysis with a mobile phase consisting of 10% water and 90% acetonitrile (v/v) at a flow rate of 0.1 mL/min [20, 21]. The reaction mixture was composed of 50 mM Tris–HCl buffer (pH 7.5), purified protein (0.08–0.40 mg/mL), and varying concentrations of CpHPG (0.1–10.0 mM). Then, 1 μL aliquot of the reaction mixture was injected and separated using a Poroshell 120 EC18 column (2.7 μm, 4.6 × 50 mm; Agilent, California, USA), and an amount of D-HPG was detected at 230 nm a wavelength at 40 °C by UV spectrophotometer. The peak of D-HPG was observed at 4.78 min (Fig. S1). A double reciprocal plot was applied to the model that defines the relationship between substrate concentration and enzyme activity, leading to the determination of the *K*_m_ and *k*_cat_ values, respectively.

### The whole-cell reaction

The whole cell reaction of the wild-type and mutants was determined for evaluating the amount of D-HPG by HPLC analysis with a mobile phase consisting of 0.1% (v/v) formic acid solution (90%) and acetonitrile (10%) at a flow rate of 0.5 mL/min. The reaction solution was composed of 50 mM Tris–HCl buffer (pH 7.5), 50 mM DL-HPH, 1.0 mM MnCl_2_, 1.0 mM DTT, and *E. coli* MG1655(DE3) harboring plasmid pYB1s-Hase (OD_600_, 30), and incubated at 50 ℃ with 200 rpm rotation for 1.0 h. Subsequently, the expressed cells of *E. coli* BL21(DE3) with plasmid pET-28a( +)-Case or variants (final OD_600_ = 20) were resuspended, and the reaction was carried out at different time intervals 0 h, 2 h, 6 h, 10 h, and 18 h. The reaction mixture was then centrifuged at 10000 rpm for 5 min. Then, 5 μL aliquot of the reaction mixture was injected and separated using an ECLIPSE PLUS C18 column (5 μm, 4.6 × 50 mm, Agilent, California, USA), the amount of D-HPG was detected at 230 nm wavelength at 40 °C by UV spectrophotometer.

### LC–MS analysis

The product was further analyzed by Liquid Chromatograph Mass Spectrometer (LC–MS) with a C18 column (250 mm × 4.6 mm, 5 μm). The detection condition was set as follows: the mobile phase included 0.1% (v/v) formic acid solution (90%) and acetonitrile (10%), the flow rate was 0.5 mL/min, ultraviolet detection wavelength was set at 230 nm, the column temperature was kept at 40 °C, and the injection volume was 5 μL. LC–MS was performed with an electrospray ion source (ESI) and the method of negative ion detection. The scanning range was set from *m/z* 50 to 500. The interface temperature was 350 °C, and the desolation line temperature was set at 250 °C with an atomizer flow of 0.5 L/min. The heating block temperature was kept at 200 °C.

### Docking of substrate into DCase

The crystal structure of the DCase enzyme (PDB ID: 1ERZ) was retrieved from protein databases [22]. The CpHPG compound information was searched using the CAS number 68780–35-8 on the PubChem website (https://pubchem.ncbi.nlm.nih.gov/). The structure of CpHPG was obtained by conducting the energy minimization using CHEM3D software (Version 20.0). The ligand molecules of CpHPG were docked into DCase protein using Autodock software (Version 4.2) [9].

### Molecular dynamics (MD) simulation

Molecular dynamics simulation was performed by using the Gromacs 2022.3 software. AmberTools22 was used to add the GAFF force field for small molecule preprocessing. Moreover, Gaussian 16W was used to hydrogenate the small molecules and calculate using restrained electrostatic potential (RESP) approach. Potential data was added to the topology file of the molecular dynamics system. The simulation conditions were adjusted at static temperature of 323 K and atmospheric pressure (1 Bar). Amber99sb-ildn was used as a force field, and water molecules were used as a solvent (Tip3p water model). The total charge of the simulation system was neutralized by adding an appropriate number of Na^+^ ions. The simulation system adopts the steepest descent method to minimize the energy. The isothermal isovolumic ensemble (NVT) equilibrium and isothermal isobaric ensemble (NPT) equilibrium were carried out for 100000 steps with the 0.1 ps coupling constant at 100 ps duration. Finally, the free molecular dynamics simulation was performed. The process consisted of 5000000 steps, the step length was 2 fs, and the total duration was around 100 ns. The built-in tool software was used to analyze the trajectory, and the root-mean-square variance (RMSD) and other data were calculated.

## Results

### Site-directed mutagenesis and protein purification

Six plasmids with single-point mutation [pET-28a(+)-Case-R3E, pET-28a(+)-Case-Q4C, pET-28a(+)-Case-T212S, pET-28a(+)-Case-A302Y, pET-28a(+)-Case-A302C and pET-28a(+)-Case-E303Q] were constructed according to the protocol of QuikChange® Site-Directed Mutagenesis Kit (Stratagene, USA). Plasmid pET-28a(+)-Case and 6 mutants were overexpressed in *E. coli* BL21(DE3) and purified to apparent homogeneity by Ni–NTA affinity chromatography. SDS-PAGE analysis revealed that molecular weight of all purified proteins with an N&C-terminal His_6_-tag was about 37 kDa, which is consistent with the calculated molecular mass of DCase and its mutants (Fig. S2).

### Biochemical characterization

#### Relative activity

**Fig. 2:**
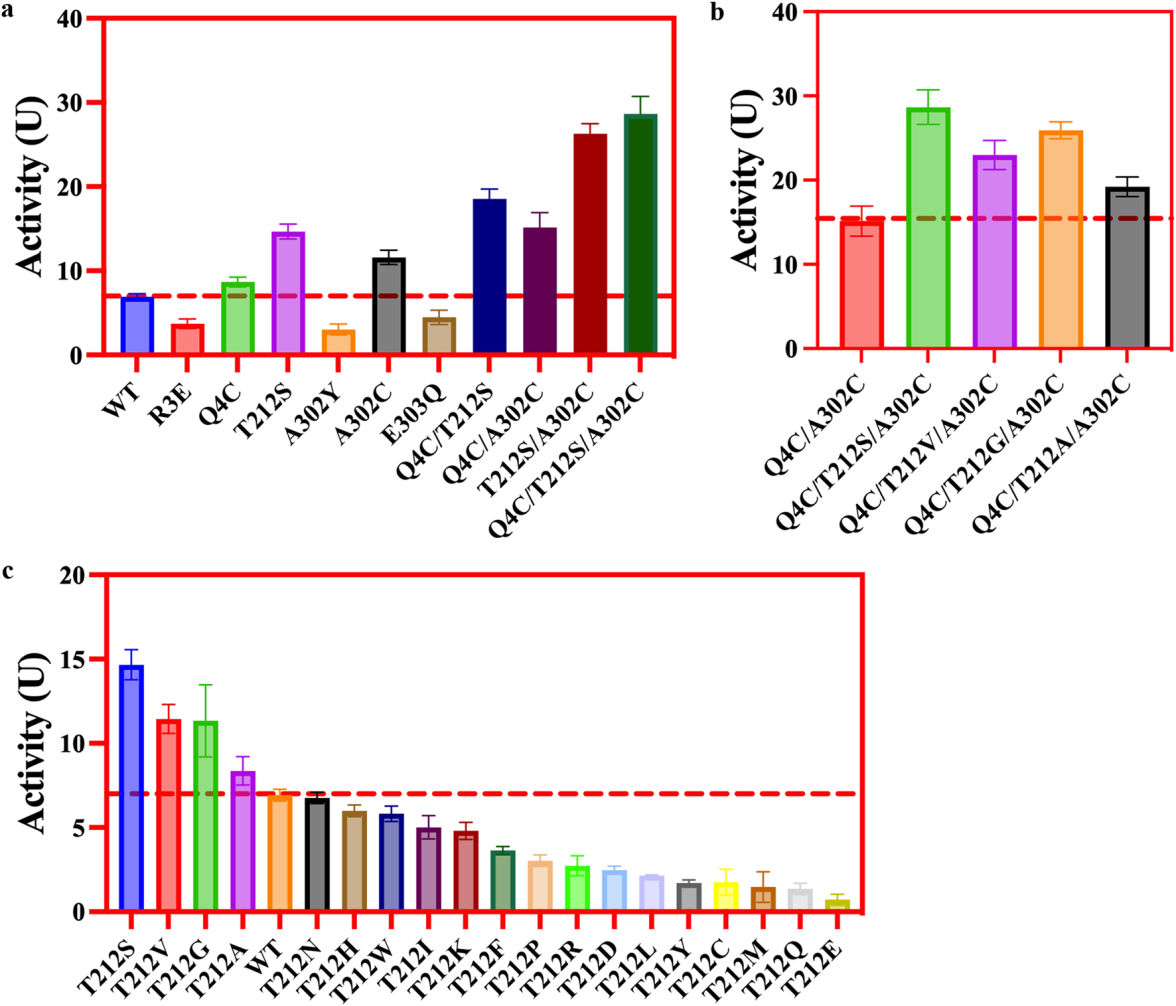
The activity data of D-HPG by site mutation **a** Single point mutations and combinatorial mutations **b** Triple-point mutants of top-mid-end structure **c** T212-site saturation mutation

Among 6 single-point mutants (R3E, Q4C, T212S, A302Y, A302C, E303Q), the relative activity of 3 mutants (Q4C, 8.45 ± 1.26 U; T212S, 14.65 ± 2.56 U; and A302C, 11.56 ± 2.22 U) was high than the wild-type (7.00 ± 0.81 U), which was about 1.21, 2.09 and 1.65-fold of the wild-type, respectively (Fig. 2a).

Based on the relative activity data of 6 single-point mutants, 3 single-point mutants (Q4C, T212S, A302C) with high relative activity were used as PCR templates and 3 double and 1 triple-point mutants (Q4C/T212S, Q4C/A302C, T212S/A302C, and Q4C/T212S/A302C) were constructed. Interestingly, the activities of 3 double-point mutants were much higher than single-point mutants (Fig. 2a). In particular, the activity of the triple-point mutant Q4C/T212S/A302C reached about 29.77 ± 4.52 U, which was about 4.25-fold higher than wild-type (Fig. 2a).

It was hypothesized that the triple-point mutant Q4C/T212S/A302C might disrupt the epistatic effect (top-mid-end structure) that leads to an increase in enzymatic activity [10, 23, 24]. To further verify this hypothesis, the single-point mutant T212S with high relative activity was selected as the PCR template for subsequent mutant construction by site-saturation mutagenesis. As compared to the wild-type, 3 beneficial mutants (T212V, T212G, and T212A) were screened out from the saturated mutants at residue 212 (Fig. 2c). Subsequently, the enzymatic assay showed that the relative activities of three top-mid-end combination mutants (triple-point mutants: Q4C/T212V/A302C, Q4C/T212G/A302C, and Q4C/T212A/A302C) were higher than double-point mutant Q4C/A302C but lower than mutant Q4C/T212S/A302C (Fig. 2b). These results were consistent with the triple-point mutant Q4C/T212S/A302C, confirming the hypothesis about the epistatic effect.

### Thermostability

The purified proteins of 6 mutants (Q4C, A302C/Q4C, Q4C/T212S/A302C, Q4C/T212V/A302C, Q4C/T212G/A302C, Q4C/T212A/A302C) and wild-type were incubated at 60 °C and determined the half-life. 6 mutants exhibited significant improvement in thermal stability as shown in Table 1. The half-life of 1 double and 4 triples-point mutants was about 2.19–2.69 times of wild-type (28.07 ± 2.21 min). Half-life of mutant Q4C/A302C (61.65 ± 3.72 min) was about 2.20-fold of WT, and 1.48-fold of Q4C (41.42 ± 3.74 min). This high thermostability could be mainly explained by the data source. Some experimental data was collected at high temperatures, which might be learned by the neural network during feature extraction.

**Table 1:** Comparison of the properties of the wild-type and variants

	WT	Q4C	Q4C/A302C	Q4C/T212S/A302C	Q4C/T212V/A302C	Q4C/T212G/A302C	Q4C/T212A/A302C
Stability (60 °C)							
T1/2 (min)	28.07 ± 2.21	41.42 ± 3.74	61.65 ± 4.33	63.24 ± 3.25	58.83 ± 3.27	75.44 ± 3.11	70.14 ± 4.25
Activity (65 °C)							
Activity (U/mg)	7.07 ± 0.43	9.43 ± 1.27	15.43 ± 3.64	29.77 ± 2.22	23.97 ± 4.65	25.98 ± 3.25	19.97 ± 3.31
*K*_m_ (mM)	2.59 ± 0.22	2.44 ± 0.46	2.56 ± 0.15	2.12 ± 0.26	2.19 ± 0.26	2.34 ± 0.26	2.38 ± 0.62
*k*_cat_ (min ^−1^)	479.01 ± 12.74	753.62 ± 22.29	1754.83 ± 43.98	2288.27 ± 39.51	1973.27 ± 39.51	2199.27 ± 39.51	1824.65 ± 42.16
*k*_cat_/*K*_m_ (min^−1^⋅mM^−1^)	184.94 ± 13.05	308.86 ± 15.91	685.17 ± 30.99	1079.37 ± 43.33	901.89 ± 72.10	939.85 ± 56.33	766.66 ± 34.29

### Kinetic parameters

The kinetic parameters of wild-type and 6 mutants were analyzed and shown in Table 1. All proteins had similar *K*_m_ value and 6 mutations showed a substantial positive impact on the enzyme activity towards CpHPG. All 6 mutants exhibited about a 1.57–4.78-fold increase in the apparent *k*_cat_ value and about a 1.67–5.84-fold increase in catalytic efficiency *k*_cat_/*K*_m_ as compared to WT (*k*_cat_, 479.01 ± 12.74 min^−1^; *k*_cat_/*K*_m_, 184.94 ± 13.05 min^−1^⋅mM^−1^). Especially, the* k*_cat_/*K*_m_ of triple-point mutant Q4C/T212S/A302C was about 1079.37 ± 43.33 min^−1^⋅mM^−1^ for CpHPG, which exhibited about 5.84-fold improvement in *k*_cat_/*K*_m_ value as compared to wild-type. These results indicated that the combination of three-site mutations exerted a significant cumulative effect on the improvement of the enzyme catalytic efficiency for DCase.

### Microbial production of D-HPG by the whole-cell reaction

**Fig. 3:**
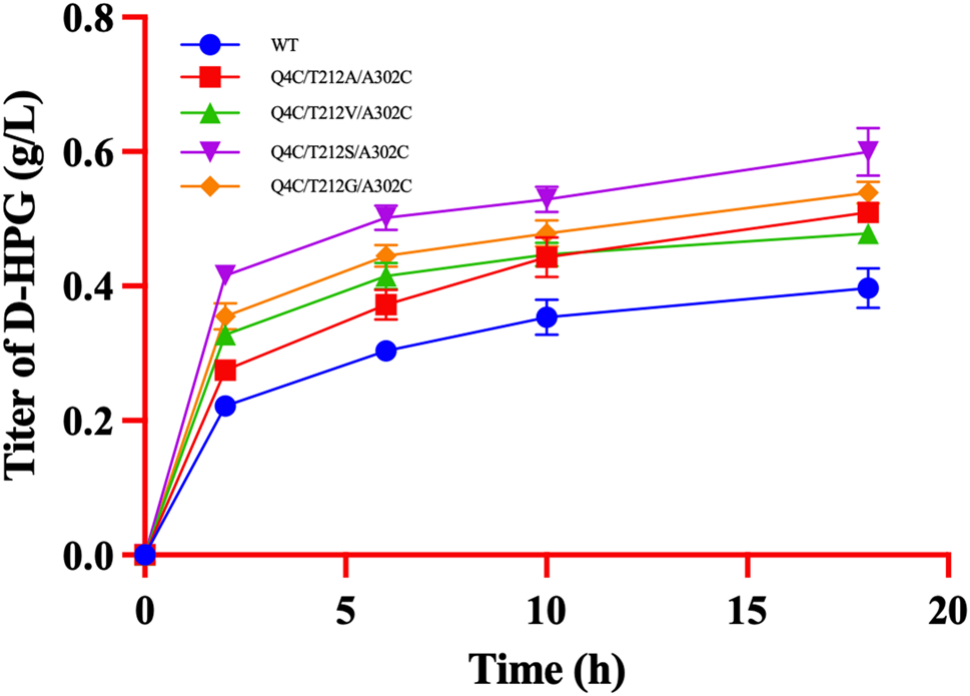
Production of D-HPG by the whole cell reaction. The substrate concentrations of DL-HPH were 50 mM

The effect of wild-type and 4 triple-point mutants on the titer of D-HPG was investigated by the whole-cell reaction method. The reaction temperature was set at 50 ℃ under similar conditions. The reaction products of wild-type vs. mutant gradually increased with time, and mutant Q4C/T212S/A302C produced the highest titer of D-HPG (0.43 ± 0.07 g/L at 2 h and 0.60 ± 0.04 g/L at 18 h), which was about 2.04-fold and 1.50-fold of wild-type (0.21 ± 0.05 g/L at 2 h and 0.40 ± 0.08 g/L at 18 h) (Fig. 3).

The production rate of D-HPG was further verified by LC–MS. The molecular weight of D-HPG is 167.1, and the molecular weight of the enzymatic product was about 168.1 under positive ion detection, which showed the same as the authentic standard D-HPG. The typical fragment ion peaks with molecular weights of 150 and 334.1 were also identical (Fig. S3).

### Structural interpretation of the improved activity

**Fig. 4:**
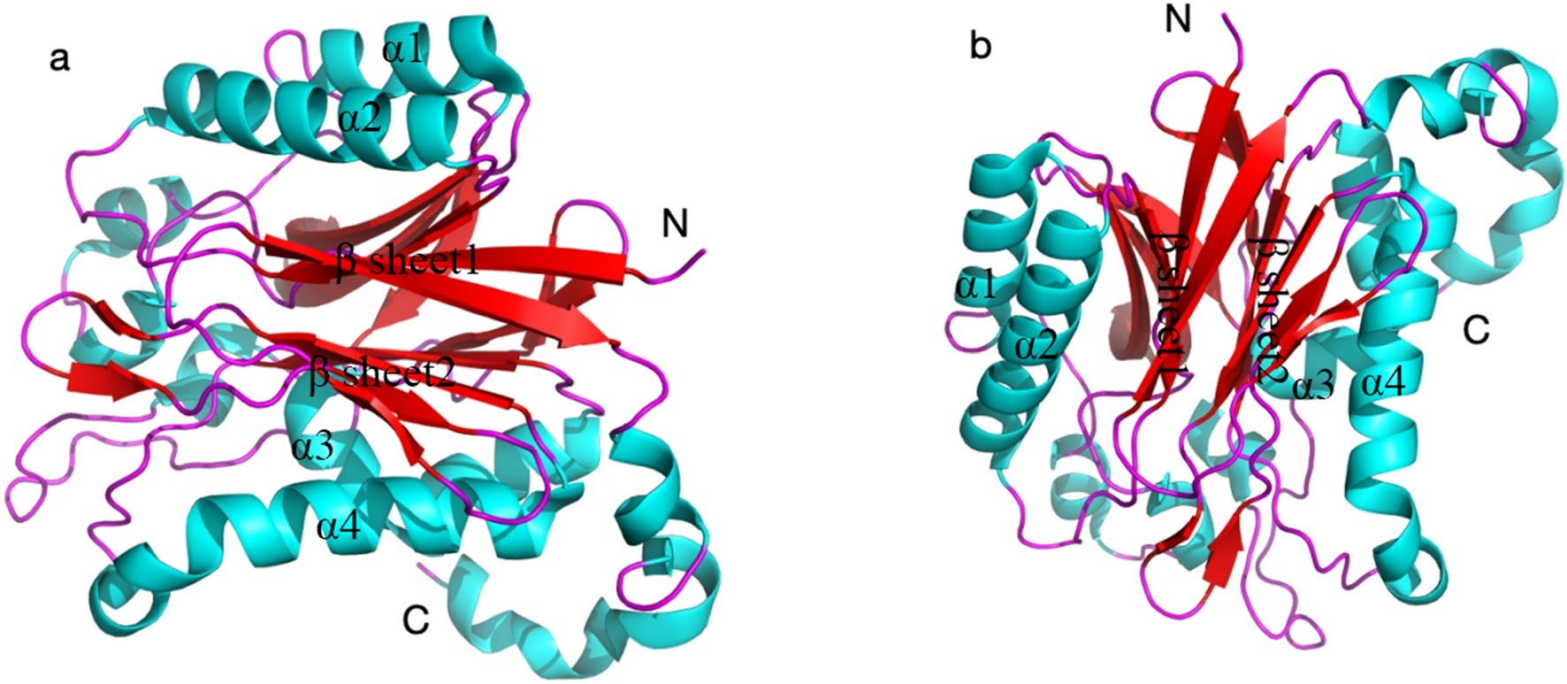
Horizontal and Vertical DCase structure **a** Horizontal structure of DCase **b** Vertical structure of DCase

The monomer of DCase exhibits a four-layer structure with two layers of α-helices and two layers of β-sheets, which are flanked by helices α1 and α3 on one side, and helices α5 and α6 on the other side (Fig. 4).

**Fig. 5:**
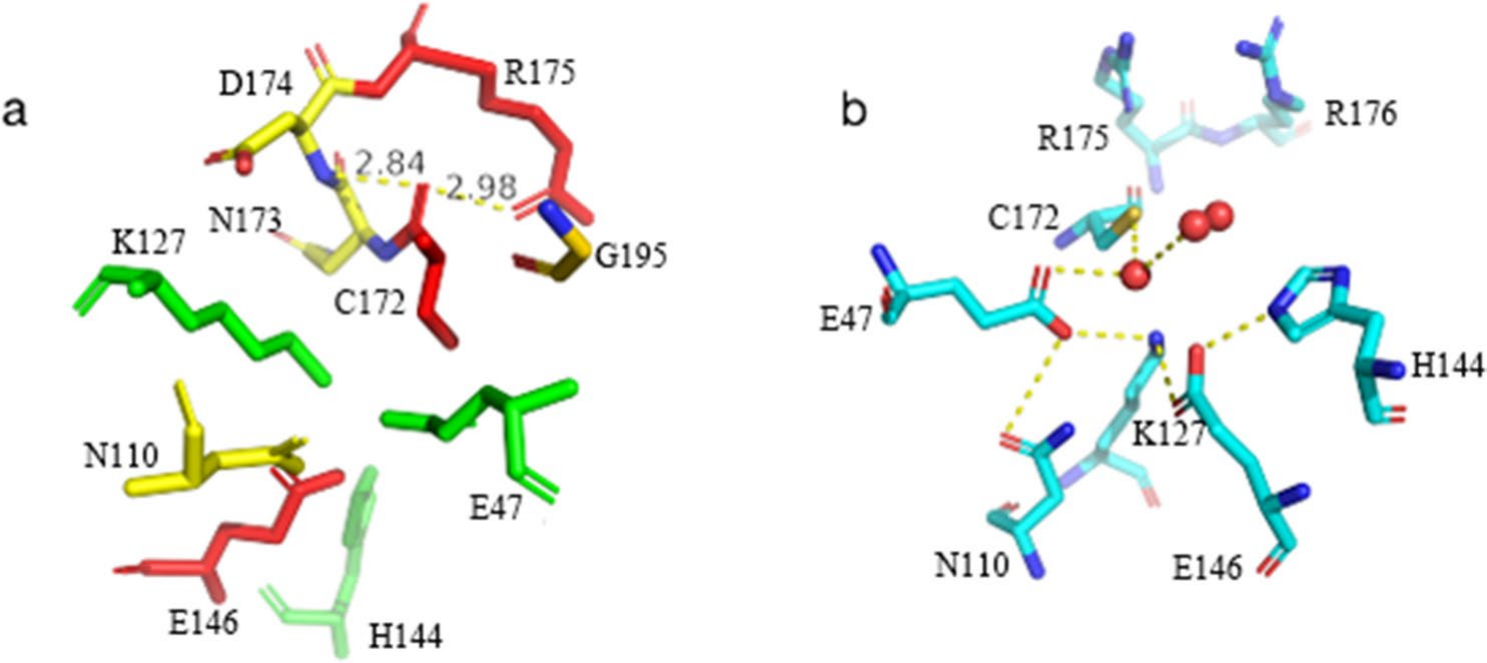
Structure modeling of the active pocket in DCase. **a** The catalytic pocket of DCase and DCase-M3. **b** Residue C172 and the surrounding residues

The β-folded sheets are divided into two strands with six β-folded sheets per strand and show continuous sequence arrangement. Tightly packed hydrophobic side chains form the interfaces between the β-folded sheets. The helices and sheets show hydrophobic interaction among amino acid residue chians. The catalytic residue C172 is located at the edge of the central β-sheet in the crystal structure. The carbonyl oxygen of C172 forms hydrogen bonds with the amide group of D174 (2.84 Å) and guanidinium group of R175 (2.98 Å), which maintained the conformational stablility of carbon skeleton and the orientation of the side chain of residue C172 (Fig. 5).

The catalytic residues E47, K127, and C172 of DCase are located at the edges of the β-folded sheet for stabilizing the active site geometry (Fig. 5) [22]. Residue C172 is surrounded by 6 residues E47, K127, H144, E146, R175 and R176. The catalytic residues of DCase (E47, K127, and C172) from *Agrobacterium* sp. strain KNK712 shows the similar geometries to DHase (D51, K144, and C177) [22]. The putative catalytic triad of Glu (Asp)-Lys-Cys might be crucial for the hydrolytic function of N-carbamoylamide [22]. Xu et al. proposed that the loop region C209-Y219 acts as a active-site lid for controlling substrate access. The opening of the active site cap significantly improved the substrate accessibility and catalytic efficiency [25].

**Fig. 6:**
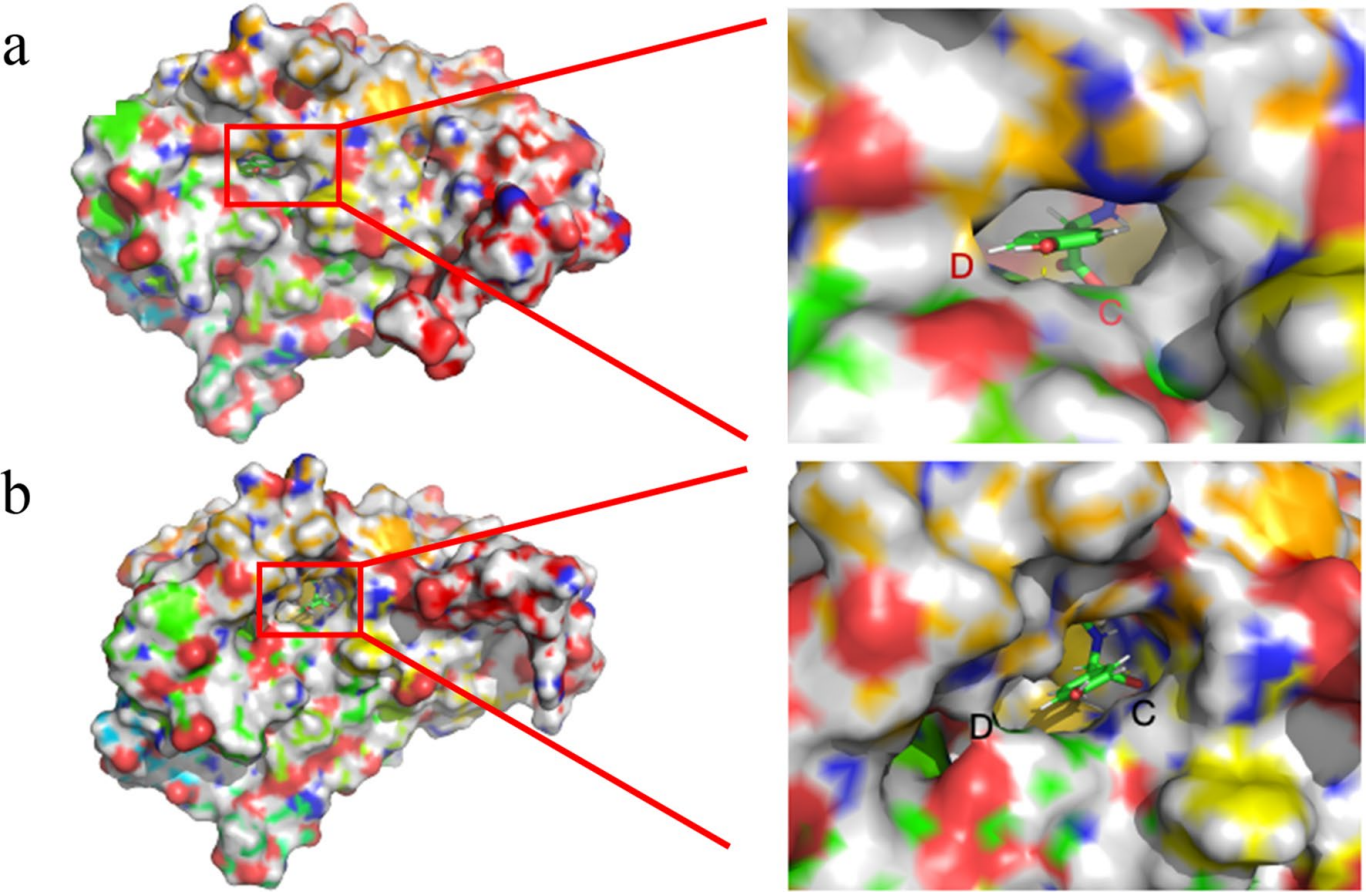
Substrate channels and the enlargement view of partial region C and D. **a** DCase (PDB ID:1erz), **b** DCase-3

In this study, as compared to crystal structure of DCase (PDB ID: 1ERZ), the overall conformation of mutant DCase-M3 (Q4C/T212S/A302C) was more compact due to a certain extent of broadening in C and D regions (Fig. 6b). On the contrary, the D region of the wild-type was relatively relaxed and C region was compact to some extent (Fig. 6a). This might be due to the expansion of C and D regions of the mutant DCase-M3, which increases the rate of substrate entry or exit, thereby accelerating the catalytic efficiency of enzymatic reaction.

**Fig. 7:**
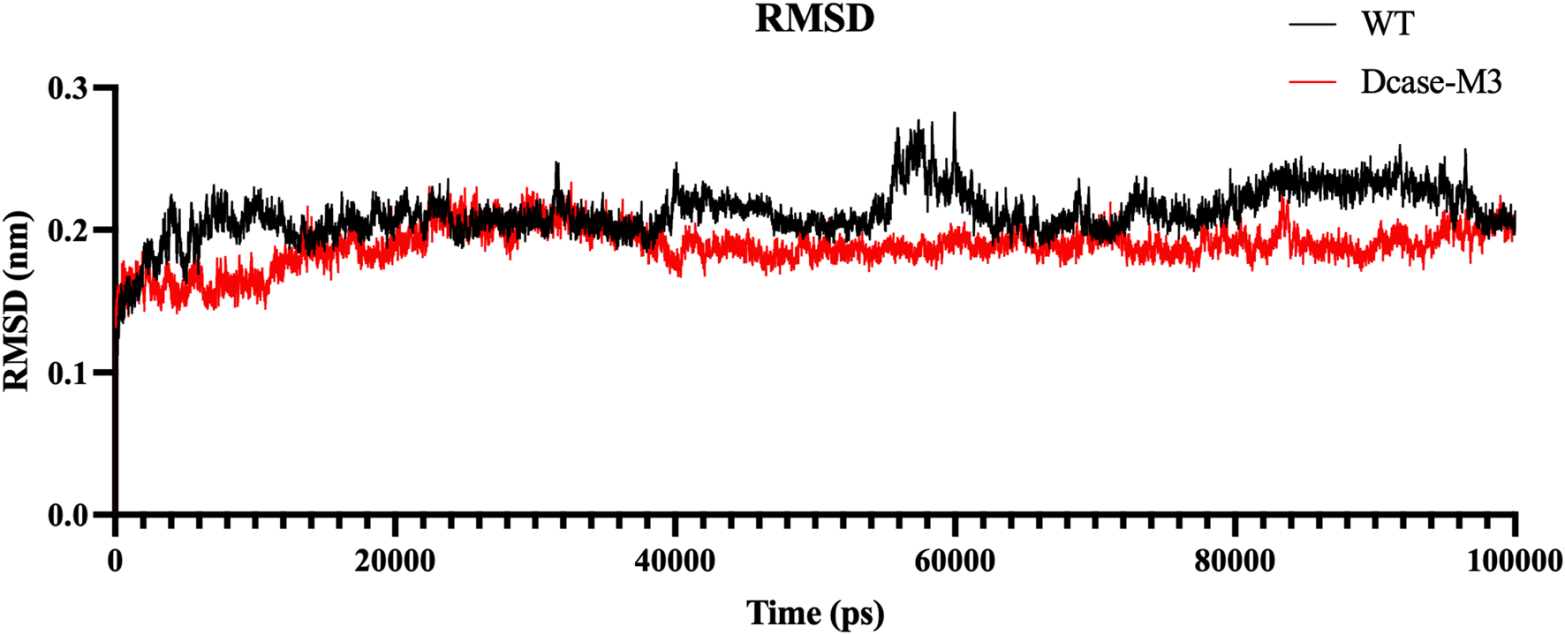
RMSD of DCase and mutant DCase-M3 with the small molecule CpHPG. RMSD of 323 K (50 °C)

In addition, molecular dynamics (MD) simulation analysis revealed that the compact structure enhanced the rigidity of the active pocket of mutant DCase-M3 as compared to wild-type. RMSD is commonly used as a metric to assess the structural stability of proteins. After MD simulation, the RMSDs of wild-type and mutant DCase-M3 almost showed similar trends, but the overall RMSD value of mutant DCase-M3 was lower than of wild-type, indicating that the structure of DCase-M3 protein was relatively more stable (Fig. 7).

**Fig. 8:**
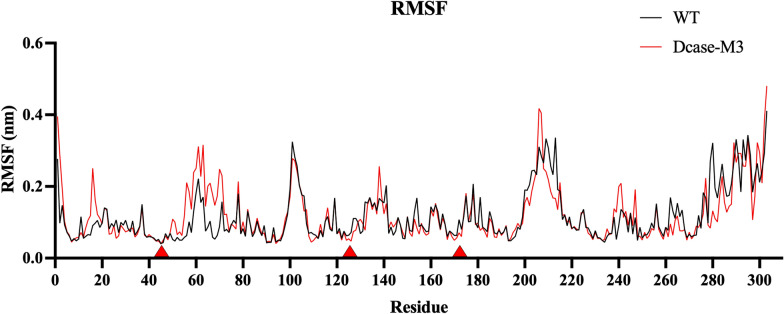
MD simulations on DCase and DCase-M3 with the small molecule CpHPG. RMSF of 323 K (50 °C)

The fluctuation rates (root mean squared fluctuation, RMSF) of DCase and DCase-M3 with the small molecule CpHPG at 323 K were also simulated by molecular dynamics. Figure 8 shows the RMSFs curves of DCase-M3 vs wild-type and RMSFs of catalytic residues (E47, K127, and C172) were much smaller than non-catalytic residues (Fig. 8).

The fluctuations in the catalytic residues and neighboring residues (within 4 Å) of DCase-M3 are also relatively less volatile as compared to wild-type, which is consistent with Yang & Bahar's study that the positional fluctuations of catalytic residues are significantly lower than other residues [26]. Enzymatic activity is associated with the low translational mobility of the catalytic residues, which assisted in maintaining the fine-tuned catalytic structure [14, 26].

**Fig. 9:**
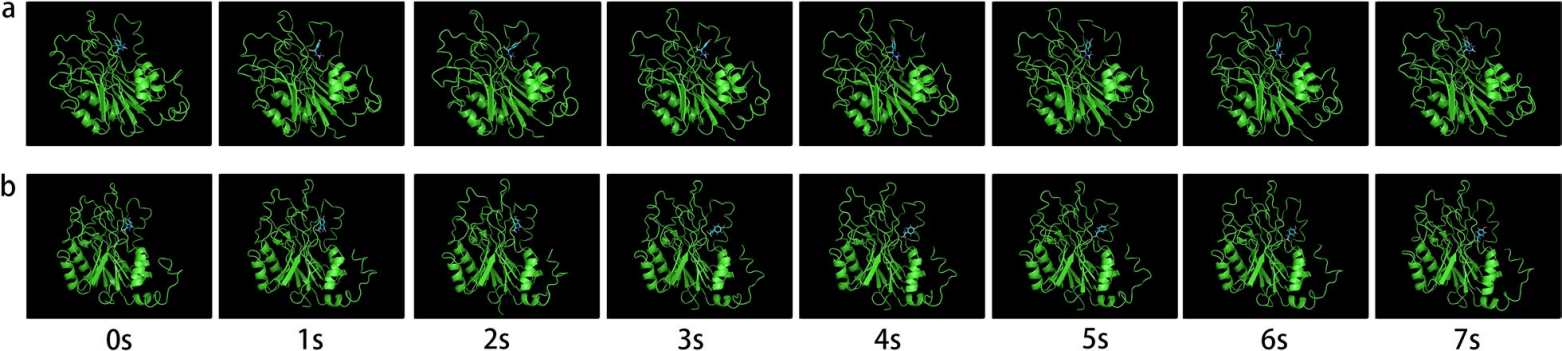
Reaction process of wild-type and DCase-M3 MD simulations. **a** wild-type **b** DCase-M3

Figure 9 shows the reaction process of wild-type and DCase-M3 catalyzing the formation of small molecule D-HPG under MD simulations (Fig. 9). DCase-M3 was observed to complete one cycle and release D-HPG within 5 s, while the wild-type did not release any small molecule D-HPG at this time. This indicates that DCase-M3 participates in a faster catalytic reaction rate by revealing a unique mechanism for regulation of distance between the substrate and key residues of the active center. These findings provide new insights into the catalytic mechanism of DCase-M3 and offer clues for further studies of the functional properties of this enzyme.

## Discussion

In recent decades, directed evolution methods such as random mutation, site-directed mutagenesis, and DNA recombination have been widely used for modification, optimization and screening of the mutated enzymes with desired properties [16, 27–31]. Enzyme-directed evolution is considered an ideal approach and is categorized into non-rational design, semi-rational design, and rational design. The non-rational or random design [32–35], involves the protein engineering through trial-and-error methods without specific theoretical guidance such as error-prone PCR [36, 37]. Semi-rational design [38–41], which falls in between non-rational and rational design. Based on the known protein structure and function, protein was modified and designed using computer simulation and other techniques. Rational design [42–44], which is based on in-depth theoretical knowledge and computer simulations for prediction of protein structure and modification, such as molecular dynamics simulations and machine learning techniques, allowing for reasonable prediction of protein properties. Rational design methods are further categorized into white-box and black-box models (Fig. S4). The white-box model involves the visual observation of protein-small molecule interactions through molecular docking and molecular dynamics simulation, while the black-box model only requires input data to generate results without knowledge of the intermediate processes, such as machine learning and deep learning. However, these methods are cumbersome, expensive, and time-consuming. There is an urgent need to develop an efficient approach for enzyme-directed evolution.

Currently, two deep learning strategies have been reported for *k*_cat_ prediction, such as Deeplearning approach and Turnover Number Prediction model (TurNup) [45, 48]. Based on the BRENDA and SABIO-RK enzyme databases, deep learning approach was developed for *k*_cat_ prediction using substrate structures and protein sequences as inputs [45]. The model has been demonstrated to be able to predict *k*_cat_ values on a large scale in various organisms and identify key residues that have effect on protein properties [45–47]. However, Deep learning approach only calculate one protein sequence with one substrate at a time and does not account for the effects of environmental factors such as pH and temperature [45]. Kroll et al. reported a turnover number prediction (TurNuP) method for *k*_cat_ values prediction for the uncharacterized data of wild-type enzymes using machine and deep learning [48]. This method was applied to enzymes with less than 40% protein similarity in the training set [48]. However, the prediction accuracy of TurNuP was almost the same as the experimental estimation, and the model accuracy needs to be further improved [48].

To overcome these shortcomings, “Feitian” software was developed througn 4 rounds of iterations and upgrades. Learning from the strategy of deeplearning model, “Feitian” version 1.0 automatically executes the saturated mutation of all residues of one protein, assisted in the prediction of *k*_*cat*_ values (K1) of all mutations, and storage of output results as tsv file. “Feitian” version 2.0 was integrated from the “TurNuP” model and the deeplearning model of “Feitian” version 1.0, and the *k*_*cat*_ values (K2) were predicted [49–51]. However, data analysis revealed that K1 (calculated by Deep learning Approach) is significantly smaller than K2 (calculated by TurNuP), as shown in Table S4. The predicted value K2 of wild-type (*k*_cat_, 16.64) was about 3.83 times than K1 (*k*_cat_, 4.34). Therefore, the output *k*_cat_ value was updated to {*k*_cat_ (s^−1^) = [Max(*k*_cat_2/*k*_cat_1)/Min(*k*_cat_2/*k*_cat_1)]*Min(*k*_cat_2/*k*_cat_1)}. “Feitian” version 3.0 created a visual interface using the PyQt5 (version 5.15.10) technology, but it less productive due to low implementation speed and time-consuming process. “Feitian” 4.0 version corrected this deficiency and merged the enzymatic properties, such as catalytic activity, thermostability, and acted as executable software by auto-py-to-exe function of pyinstaller (version 6.3.0). The running time is about 2.74 h per 325 amino acids for one protein using Nvidia GeForce RTX3060 for calculation. This upgraded software version is more convenient for operations and can run on an ordinary laptops (Fig. S5).

In this study, software “Feitian” was applied for the first time to predict and enhance the enzymatic activity and thermal stability of N-carbamoyl-D-amino acid amidohydrolase, after prediction and screening, 3 of six predicted mutants had higher activity than the wild-type, which indicates that the prediction accuracy of “Feitian” software reached 50%. Finally, a triple-point mutant Q4C/T212S/A302C with 4.25-fold improvement in activity and 2.25-fold increase in thermal stability was successfully obtained by mutant combination. Molecular dynamics simulation analysis revealed that activity of DCase-M3 was enhanced either by changing the active pocket to remove substrate inhibition (Fig. 6) or by fine-tuning the design of the catalytic site (Fig. 9).

In conclusion, this study provides an efficient new approach “Feitian” for rational design for directed enzyme evolution. A triple-point mutant with enhanced enzymatic activity and thermostability was successfully obtained using this new approach. The prediction accuracy of “Feitian” reached about 50% in enzymatic activity. This indicates that there is still much room for improvement in algorithms and dataset by artificial intelligence.

### Supplementary Information

Supplementary Material 1: Table S1 Prediction of “Feitian”. Table S2 Prediction of “Feitian” in sites 3, 4, 212, 303 and 304. Table S3 Primers used in this study. Table S4 Prediction of “Feitian” version 1.0 and 2.0’. Fig. S1 a) HPLC data of D-HPG, CpHPG and D-HPH b) D-HPG of 0.5mM, 1.0mM and 2.0mM. Fig. S2. SDS-PAGE profile of purified D-amino acid amidohydrolase by Ni-NTA agarose. M) marker, 1) wild-type, 2) R3E, 3) Q4C, 4) T212S, 5) A302Y, 6) A302C, 7) E303Q. Fig. S3 LC-MS data of D-HPG. Fig. S4 Black-box modeling from input data to generated result. Fig. S5 “Feitian” visualization interface.

## Data Availability

The authors confirm that the data supporting the findings of this study are available within the article and its supplementary materials.
