# Correction: Improving the enzymatic activity and stability of N‑carbamoyl hydrolase using deep learning approach

**DOI:** 10.1186/s12934-024-02461-7

**Published:** 2024-07-02

**Authors:** Fa Zhang, Muhammad Naeem, Bo Yu, Feixia Liu, Jiansong Ju

**Affiliations:** 1 College of Life Science, Hebei Normal University, Shijiazhuang, 050024 China; 2 Institute of Microbiology, Chinese Academy of Sciences, Beijing, 100101 China; 3 Hebei Collaborative Innovation Center for Eco-Environment, Shijiazhuang, 050024 China

**Correction: Microbial Cell Factories (2024) 23:164**

10.1186/s12934-024-02439-5.

Following publication of the original article, the authors would like to correct the acknowledgment. The updated acknowledgment is given below and the changes have been highlighted in **bold typeface**.

The sentence currently reads:

**Acknowledgements** This work was supported by the National Natural Science Foundation of China (31,971,204), Funds for Central Guiding Local Science and Technology Development (236Z2801G), National Key R&D Program of China (2018YFA0901400).

The sentence should read:

**Acknowledgements** This work was supported by the National Natural Science Foundation of China (31,971,204), Funds for Central Guiding Local Science and Technology Development (236Z2801G) **and Heilongjiang Provincial Major Project for Technical Industrialization, China (CG22006).**

The original article has been corrected.

