# Supplementary material for: Improving the enzymatic activity and stability of N-carbamoyl hydrolase using deep learning approach
Source: Microb Cell Fact. 2024 Jun 4;23:164. doi: 10.1186/s12934-024-02439-5 (PMC11151596; doi:10.1186/s12934-024-02439-5)
Supplement: Supplementary file 1 — Supplementary Material 1: Table S1 Prediction of “Feitian”. Table S2 Prediction of “Feitian” in sites 3, 4, 212, 303 and 304. Table S3 Primers used in this study. Table S4 Prediction of “Feitian” version 1.0 and 2.0’. Fig. S1 a) HPLC data of D-HPG, CpHPG and D-HPH b) D-HPG of 0.5mM, 1.0mM and 2.0mM. Fig. S2. SDS-PAGE profile of purified D-amino acid amidohydrolase by Ni-NTA agarose. M) marker, 1) wild-type, 2) R3E, 3) Q4C, 4) T212S, 5) A302Y, 6) A302C, 7) E303Q. Fig. S3 LC-MS data of D-HPG. Fig. S4 Black-box modeling from input data to generated result. Fig. S5 “Feitian” visualization interface. [file 12934_2024_2439_MOESM1_ESM.docx]

**Supplementary Information**

**For**

**Improving the enzymatic activity and stability of N-carbamoyl hydrolase using deep learning approach**

Fa Zhang^1,2^, Muhammad Naeem^1^, Bo Yu^2^, Feixia Liu^2*^ and Jiansong Ju^1,3*^

*^1^ College of Life Science, Hebei Normal University, Shijiazhuang 050024, China*

*^2^ Institute of Microbiology, Chinese Academy of Sciences, Beijing 100101, China*

*^3^ Hebei Collaborative Innovation Center for Eco-Environment, Shijiazhuang 050024, China*

## Supplementary Table

**Table S1 Prediction of “Feitian”**

| Enzyme | OriginAA | site | MutationAA | complete | *k*_cat_ (s^-1^) |
| --- | --- | --- | --- | --- | --- |
| \| \| Seq \| \| --- \| \| Seq \| \| Seq \| \| Seq \| \| Seq \| \| Seq \| \| Seq \| \| Seq \| \| Seq \| \| Seq \| \| Seq \| \| Seq \| \| Seq \| \| Seq \| \| Seq \| \| Seq \| \| Seq \| \| Seq \| \| Seq \| \| Seq  … \| \| \| --- \| --- \| --- \| --- \| --- \| --- \| --- \| --- \| --- \| --- \| --- \| --- \| --- \| --- \| --- \| --- \| --- \| --- \| --- \| --- \| --- \| | \| M \| \| --- \| \| M \| \| M \| \| M \| \| M \| \| M \| \| M \| \| M \| \| M \| \| M \| \| M \| \| M \| \| M \| \| M \| \| M \| \| M \| \| M \| \| M \| \| M \| \| M \|   … | \| 1 \| \| --- \| \| 1 \| \| 1 \| \| 1 \| \| 1 \| \| 1 \| \| 1 \| \| 1 \| \| 1 \| \| 1 \| \| 1 \| \| 1 \| \| 1 \| \| 1 \| \| 1 \| \| 1 \| \| 1 \| \| 1 \| \| 1 \| \| 1 \|   … | \| R \| \| --- \| \| G \| \| V \| \| L \| \| I \| \| P \| \| F \| \| Y \| \| W \| \| S \| \| T \| \| C \| \| M \| \| N \| \| Q \| \| D \| \| E \| \| K \| \| A \| \| H \|   … | \| TRUE \| \| --- \| \| TRUE \| \| TRUE \| \| TRUE \| \| TRUE \| \| TRUE \| \| TRUE \| \| TRUE \| \| TRUE \| \| TRUE \| \| TRUE \| \| TRUE \| \| TRUE \| \| TRUE \| \| TRUE \| \| TRUE \| \| TRUE \| \| TRUE \| \| TRUE \| \| TRUE \|   … | \| 14.34 \| \| --- \| \| 15.68 \| \| 14.61 \| \| 18.17 \| \| 13.51 \| \| 13.92 \| \| 15.68 \| \| 14.77 \| \| 14.77 \| \| 14.34 \| \| 14.34 \| \| 21.11 \| \| 16.64 \| \| 14.77 \| \| 23.08 \| \| 15.68 \| \| 19.89 \| \| 13.51 \| \| 15.22 \| \| 13.11  … \| |

Seq represents the mutated amino acid sequence.

Input sequence: MTRQMILAVGQQGPIARAETREQVVVRLLDMLTKAASRGANFIVFPELALTTFFPRWHFTDEAELDSFYETEMPGPVVRPLFEKAAELGIGFNLGYAELVVEGGVKRRFNTSILVDKSGKIVGKYRKIHLPGHKEYEAYRPFQHLEKRYFEPGDLGFPVYDVDAAKMGMFICNDRRWPEAWRVMGLRGAEIICGGYNTPTHNPPVPQHDHLTSFHHLLSMQAGSYQNGAWSAAAGKVGMEENCMLLGHSCIVAPTGEIVALTTTLEDEVITAAVDLDRCRELREHIFNFKQHRQPQHYGLIAEL

Input Substrate Name: n-Carbamoyl-d-p-hydroxyphenylglycine

Input Substrate SMILES: C1=CC(=CC=C1N(CC(=O)O)C(=O)N)O

Input Product SMILES: C1=CC(=CC=C1C(C(=O)O)N)O

Input mask: None

**Table S2 Prediction of “Feitian” in sites 3, 4, 212, 303 and 304**

| enzyme | OriginAA | site | MutationAA | complete | *k*_cat_ (s^-1^) |
| --- | --- | --- | --- | --- | --- |
| \| \| Seq \| \| --- \| \| Seq \| \| Seq \| \| Seq \| \| Seq \| \| Seq \| \| Seq \| \| Seq \| \| Seq \| \| Seq \| \| Seq \| \| Seq \| \| Seq \| \| Seq \| \| Seq \| \| Seq \| \| Seq \| \| Seq \| \| Seq \| \|  \| \| \| --- \| --- \| --- \| --- \| --- \| --- \| --- \| --- \| --- \| --- \| --- \| --- \| --- \| --- \| --- \| --- \| --- \| --- \| --- \| --- \| --- \| | \| R \| \| --- \| \| R \| \| R \| \| R \| \| R \| \| R \| \| R \| \| R \| \| R \| \| R \| \| R \| \| R \| \| R \| \| R \| \| R \| \| R \| \| R \| \| R \| \| R \| \|  \| | \| 3 \| \| --- \| \| 3 \| \| 3 \| \| 3 \| \| 3 \| \| 3 \| \| 3 \| \| 3 \| \| 3 \| \| 3 \| \| 3 \| \| 3 \| \| 3 \| \| 3 \| \| 3 \| \| 3 \| \| 3 \| \| 3 \| \| 3 \| \|  \| | \| T \| \| --- \| \| E \| \| G \| \| A \| \| C \| \| N \| \| K \| \| S \| \| P \| \| Q \| \| V \| \| M \| \| L \| \| I \| \| F \| \| D \| \| H \| \| Y \| \| W \| | \| TRUE \| \| --- \| \| TRUE \| \| TRUE \| \| TRUE \| \| TRUE \| \| TRUE \| \| TRUE \| \| TRUE \| \| TRUE \| \| TRUE \| \| TRUE \| \| TRUE \| \| TRUE \| \| TRUE \| \| TRUE \| \| TRUE \| \| TRUE \| \| TRUE \| \| TRUE \| | \| 23.08 \| \| --- \| \| 23.08 \| \| 22.41 \| \| 22.41 \| \| 21.75 \| \| 21.75 \| \| 21.11 \| \| 20.49 \| \| 19.89 \| \| 19.31 \| \| 18.74 \| \| 18.74 \| \| 18.19 \| \| 18.19 \| \| 18.19 \| \| 17.66 \| \| 17.14 \| \| 14.34 \| \| 12.73 \| |

| enzyme | OriginAA | site | MutationAA | complete | *k*_cat_ (s^-1^) |
| --- | --- | --- | --- | --- | --- |
| \| \| Seq \| \| --- \| \| Seq \| \| Seq \| \| Seq \| \| Seq \| \| Seq \| \| Seq \| \| Seq \| \| Seq \| \| Seq \| \| Seq \| \| Seq \| \| Seq \| \| Seq \| \| Seq \| \| Seq \| \| Seq \| \| Seq \| \| Seq \| \|  \| \| \| --- \| --- \| --- \| --- \| --- \| --- \| --- \| --- \| --- \| --- \| --- \| --- \| --- \| --- \| --- \| --- \| --- \| --- \| --- \| --- \| --- \| | \| Q \| \| --- \| \| Q \| \| Q \| \| Q \| \| Q \| \| Q \| \| Q \| \| Q \| \| Q \| \| Q \| \| Q \| \| Q \| \| Q \| \| Q \| \| Q \| \| Q \| \| Q \| \| Q \| \| Q \| \|  \| | \| 4 \| \| --- \| \| 4 \| \| 4 \| \| 4 \| \| 4 \| \| 4 \| \| 4 \| \| 4 \| \| 4 \| \| 4 \| \| 4 \| \| 4 \| \| 4 \| \| 4 \| \| 4 \| \| 4 \| \| 4 \| \| 4 \| \| 4 \| \|  \| | \| R \| \| --- \| \| N \| \| T \| \| D \| \| I \| \| C \| \| L \| \| M \| \| V \| \| A \| \| F \| \| S \| \| P \| \| K \| \| G \| \| Y \| \| E \| \| H \| \| W \| | \| TRUE \| \| --- \| \| TRUE \| \| TRUE \| \| TRUE \| \| TRUE \| \| TRUE \| \| TRUE \| \| TRUE \| \| TRUE \| \| TRUE \| \| TRUE \| \| TRUE \| \| TRUE \| \| TRUE \| \| TRUE \| \| TRUE \| \| TRUE \| \| TRUE \| \| TRUE \| | \| 24.50 \| \| --- \| \| 23.46 \| \| 22.41 \| \| 22.35 \| \| 21.11 \| \| 21.11 \| \| 20.49 \| \| 20.49 \| \| 19.89 \| \| 19.86 \| \| 19.31 \| \| 19.31 \| \| 18.74 \| \| 18.74 \| \| 18.71 \| \| 18.60 \| \| 17.33 \| \| 17.14 \| \| 16.72 \| |

| enzyme | OriginAA | site | MutationAA | complete | *k*_cat_ (s^-1^) |
| --- | --- | --- | --- | --- | --- |
| \| \| Seq \| \| --- \| \| Seq \| \| Seq \| \| Seq \| \| Seq \| \| Seq \| \| Seq \| \| Seq \| \| Seq \| \| Seq \| \| Seq \| \| Seq \| \| Seq \| \| Seq \| \| Seq \| \| Seq \| \| Seq \| \| Seq \| \| Seq \| \|  \| \| \| --- \| --- \| --- \| --- \| --- \| --- \| --- \| --- \| --- \| --- \| --- \| --- \| --- \| --- \| --- \| --- \| --- \| --- \| --- \| --- \| --- \| | \| T \| \| --- \| \| T \| \| T \| \| T \| \| T \| \| T \| \| T \| \| T \| \| T \| \| T \| \| T \| \| T \| \| T \| \| T \| \| T \| \| T \| \| T \| \| T \| \| T \| \|  \| | \| 212 \| \| --- \| \| 212 \| \| 212 \| \| 212 \| \| 212 \| \| 212 \| \| 212 \| \| 212 \| \| 212 \| \| 212 \| \| 212 \| \| 212 \| \| 212 \| \| 212 \| \| 212 \| \| 212 \| \| 212 \| \| 212 \| \| 212 \| \|  \| | \| Y \| \| --- \| \| M \| \| W \| \| P \| \| C \| \| S \| \| K \| \| N \| \| F \| \| R \| \| G \| \| L \| \| H \| \| I \| \| E \| \| V \| \| Q \| \| D \| \| A \| | \| TRUE \| \| --- \| \| TRUE \| \| TRUE \| \| TRUE \| \| TRUE \| \| TRUE \| \| TRUE \| \| TRUE \| \| TRUE \| \| TRUE \| \| TRUE \| \| TRUE \| \| TRUE \| \| TRUE \| \| TRUE \| \| TRUE \| \| TRUE \| \| TRUE \| \| TRUE \| | \| 30.48 \| \| --- \| \| 26.38 \| \| 21.93 \| \| 21.75 \| \| 21.05 \| \| 19.90 \| \| 19.89 \| \| 19.31 \| \| 18.74 \| \| 18.74 \| \| 18.56 \| \| 18.19 \| \| 18.19 \| \| 18.19 \| \| 18.02 \| \| 17.66 \| \| 17.14 \| \| 14.77 \| \| 13.51 \| |

| enzyme | OriginAA | site | MutationAA | complete | *k*_cat_ (s^-1^) |
| --- | --- | --- | --- | --- | --- |
| \| \| Seq \| \| --- \| \| Seq \| \| Seq \| \| Seq \| \| Seq \| \| Seq \| \| Seq \| \| Seq \| \| Seq \| \| Seq \| \| Seq \| \| Seq \| \| Seq \| \| Seq \| \| Seq \| \| Seq \| \| Seq \| \| Seq \| \| Seq \| \|  \| \| \| --- \| --- \| --- \| --- \| --- \| --- \| --- \| --- \| --- \| --- \| --- \| --- \| --- \| --- \| --- \| --- \| --- \| --- \| --- \| --- \| --- \| | \| A \| \| --- \| \| A \| \| A \| \| A \| \| A \| \| A \| \| A \| \| A \| \| A \| \| A \| \| A \| \| A \| \| A \| \| A \| \| A \| \| A \| \| A \| \| A \| \| A \| \|  \| | \| 302 \| \| --- \| \| 302 \| \| 302 \| \| 302 \| \| 302 \| \| 302 \| \| 302 \| \| 302 \| \| 302 \| \| 302 \| \| 302 \| \| 302 \| \| 302 \| \| 302 \| \| 302 \| \| 302 \| \| 302 \| \| 302 \| \| 302 \| \|  \| | \| Y \| \| --- \| \| C \| \| L \| \| F \| \| V \| \| I \| \| W \| \| T \| \| M \| \| H \| \| N \| \| R \| \| S \| \| D \| \| E \| \| K \| \| Q \| \| G \| \| P \| | \| TRUE \| \| --- \| \| TRUE \| \| TRUE \| \| TRUE \| \| TRUE \| \| TRUE \| \| TRUE \| \| TRUE \| \| TRUE \| \| TRUE \| \| TRUE \| \| TRUE \| \| TRUE \| \| TRUE \| \| TRUE \| \| TRUE \| \| TRUE \| \| TRUE \| \| TRUE \| | \| 19.89 \| \| --- \| \| 19.89 \| \| 18.74 \| \| 18.74 \| \| 17.66 \| \| 17.66 \| \| 17.66 \| \| 17.66 \| \| 17.66 \| \| 17.66 \| \| 16.68 \| \| 16.64 \| \| 16.64 \| \| 16.64 \| \| 16.64 \| \| 16.29 \| \| 16.15 \| \| 16.14 \| \| 15.03 \| |

| enzyme | OriginAA | site | MutationAA | complete | *k*_cat_ (s^-1^) |
| --- | --- | --- | --- | --- | --- |
| \| \| Seq \| \| --- \| \| Seq \| \| Seq \| \| Seq \| \| Seq \| \| Seq \| \| Seq \| \| Seq \| \| Seq \| \| Seq \| \| Seq \| \| Seq \| \| Seq \| \| Seq \| \| Seq \| \| Seq \| \| Seq \| \| Seq \| \| Seq \| \|  \| \| \| --- \| --- \| --- \| --- \| --- \| --- \| --- \| --- \| --- \| --- \| --- \| --- \| --- \| --- \| --- \| --- \| --- \| --- \| --- \| --- \| --- \| | \| E \| \| --- \| \| E \| \| E \| \| E \| \| E \| \| E \| \| E \| \| E \| \| E \| \| E \| \| E \| \| E \| \| E \| \| E \| \| E \| \| E \| \| E \| \| E \| \| E \| \|  \| | \| 303 \| \| --- \| \| 303 \| \| 303 \| \| 303 \| \| 303 \| \| 303 \| \| 303 \| \| 303 \| \| 303 \| \| 303 \| \| 303 \| \| 303 \| \| 303 \| \| 303 \| \| 303 \| \| 303 \| \| 303 \| \| 303 \| \| 303 \| \|  \| | \| R \| \| --- \| \| N \| \| T \| \| D \| \| I \| \| C \| \| L \| \| M \| \| V \| \| A \| \| F \| \| S \| \| P \| \| K \| \| G \| \| Y \| \| E \| \| H \| \| W \| | \| TRUE \| \| --- \| \| TRUE \| \| TRUE \| \| TRUE \| \| TRUE \| \| TRUE \| \| TRUE \| \| TRUE \| \| TRUE \| \| TRUE \| \| TRUE \| \| TRUE \| \| TRUE \| \| TRUE \| \| TRUE \| \| TRUE \| \| TRUE \| \| TRUE \| \| TRUE \| | \| 19.31 \| \| --- \| \| 18.74 \| \| 18.74 \| \| 18.19 \| \| 18.19 \| \| 17.66 \| \| 16.64 \| \| 16.52 \| \| 16.45 \| \| 15.68 \| \| 15.41 \| \| 15.22 \| \| 15.11 \| \| 14.77 \| \| 14.77 \| \| 14.72 \| \| 14.49 \| \| 14.49 \| \| 13.51 \| |

**Table S3 Primers used in this study**

| **Primers** | **Sequence（5’-3’）** |
| --- | --- |
| 3/4_F  R3E_F | ATTCTGGCCGTTGGTCAGCAG  CGGCCAGAATCATCTGCTCGGT |
| Q4C_F  T212A_F  T212A_R  A302C_F  A302Y_F  E303Q_F  302/303_R | CGGCCAGAATCATACAACGGGT  CGCAGCATGATCATCTGGCCAGC  CAGATGATCATGCTGCGGAACCG  GCATTATGGTCTGATTTGTGAACTG  GCATTATGGTCTGATTTATGAACTG  GCATTATGGTCTGATTGCCCAGCTG  CAGACCATAATGCTGCGGCTGAC |

**Table S4 Prediction of “Feitian” version 1.0 and 2.0**

| K1 | enzyme | OriginAA | site | MutationAA | complete | K2 |
| --- | --- | --- | --- | --- | --- | --- |
| \| 3.69 \| \| --- \| \| 2.17 \| \| 3.81 \| \| 4.74 \| \| 3.27 \| \| 2.93 \| \| 2.89 \| \| 2.57 \| \| 3.41 \| \| 2.37 \| \| 3.08 \| \| 3.8 \| \| 4.34 \| \| 2.71 \| \| 4.6 \| \| 3 \| \| 2.3 \| \| 2.65 \| \| 3.52 \| \| 1.96  … \| | \| \| Seq \| \| --- \| \| Seq \| \| Seq \| \| Seq \| \| Seq \| \| Seq \| \| Seq \| \| Seq \| \| Seq \| \| Seq \| \| Seq \| \| Seq \| \| Seq \| \| Seq \| \| Seq \| \| Seq \| \| Seq \| \| Seq \| \| Seq \| \| Seq  … \| \| \| --- \| --- \| --- \| --- \| --- \| --- \| --- \| --- \| --- \| --- \| --- \| --- \| --- \| --- \| --- \| --- \| --- \| --- \| --- \| --- \| --- \| | \| M \| \| --- \| \| M \| \| M \| \|  \| \| M \| \| M \| \| M \| \| M \| \| M \| \| M \| \| M \| \| M \| \| M \| \| M \| \| M \| \| M \| \| M \| \| M \| \| M \| \| M \| \| M  … \| | \| 1 \| \| --- \| \| 1 \| \| 1 \| \| 1 \| \| 1 \| \| 1 \| \| 1 \| \| 1 \| \| 1 \| \| 1 \| \| 1 \| \| 1 \| \| 1 \| \| 1 \| \| 1 \| \| 1 \| \| 1 \| \| 1 \| \| 1 \| \| 1  … \| | \| R \| \| --- \| \| G \| \| V \| \| L \| \| I \| \| P \| \| F \| \| Y \| \| W \| \| S \| \| T \| \| C \| \| M \| \| N \| \| Q \| \| D \| \| E \| \| K \| \| A \| \| H  … \| | \| TRUE \| \| --- \| \| TRUE \| \| TRUE \| \| TRUE \| \| TRUE \| \| TRUE \| \| TRUE \| \| TRUE \| \| TRUE \| \| TRUE \| \| TRUE \| \| TRUE \| \| TRUE \| \| TRUE \| \| TRUE \| \| TRUE \| \| TRUE \| \| TRUE \| \| TRUE \| \| TRUE  … \| | \| 14.34 \| \| --- \| \| 15.68 \| \| 14.34 \| \| 13.11 \| \| 13.51 \| \| 13.92 \| \| 15.68 \| \| 14.77 \| \| 14.77 \| \| 14.34 \| \| 14.34 \| \| 21.11 \| \| 16.64 \| \| 14.77 \| \| 23.08 \| \| 15.68 \| \| 19.89 \| \| 13.51 \| \| 15.22 \| \| 13.11  … \| |

## Supplementary figures


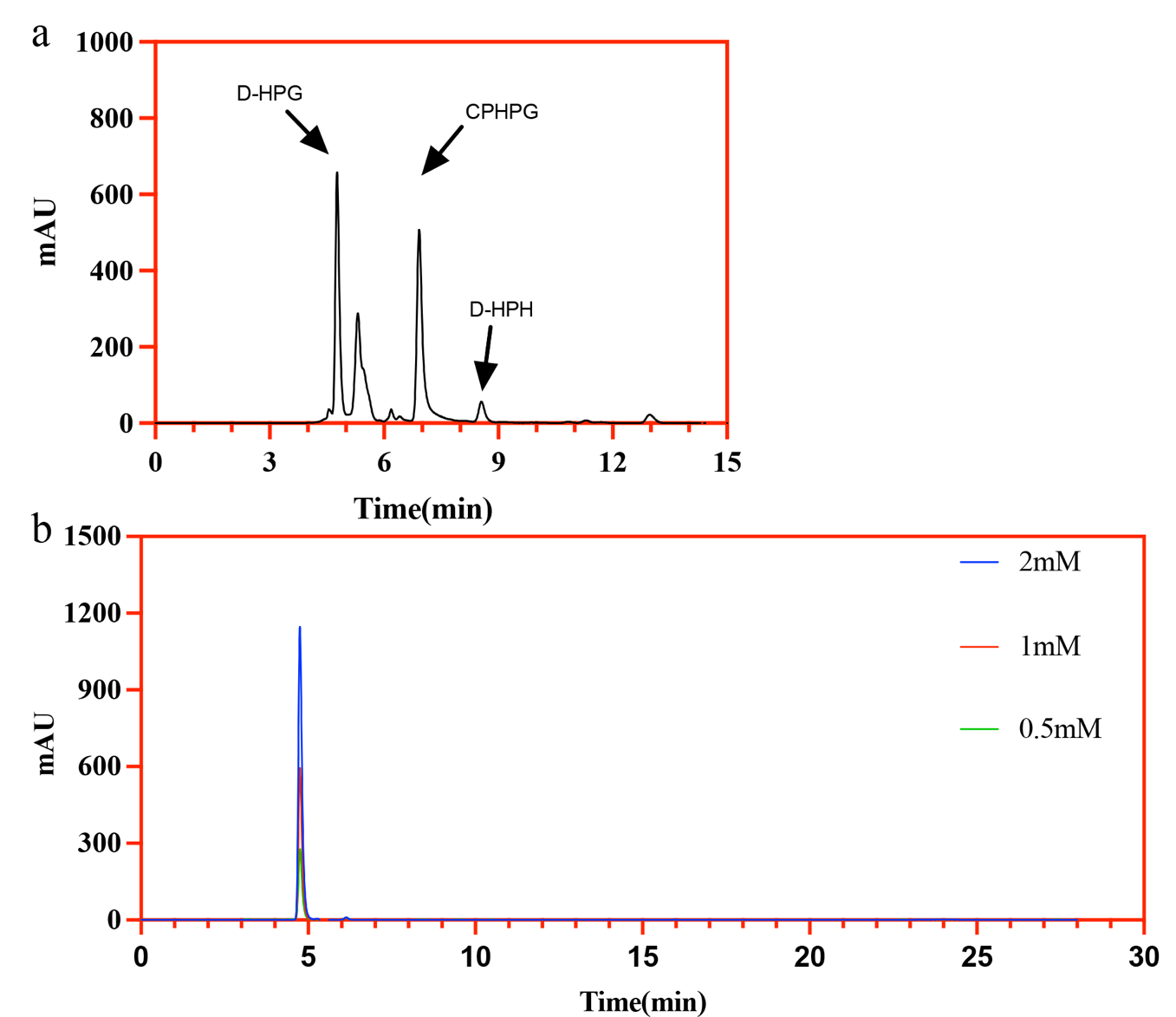


**Fig. S1** a) HPLC data of D-HPG, CpHPG and D-HPH b) D-HPG of 0.5mM, 1.0mM and 2.0mM





**Fig. S2.** SDS-PAGE profile of purified D-amino acid amidohydrolase by Ni-NTA agarose. M) marker, 1) wild-type, 2) R3E, 3) Q4C, 4) T212S, 5) A302Y, 6) A302C, 7) E303Q.


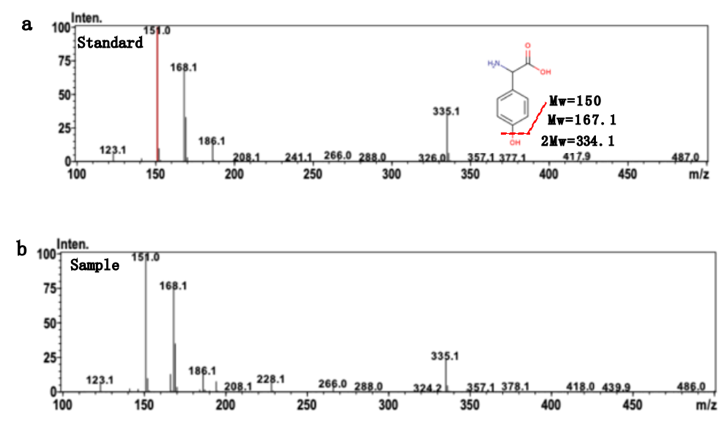


**Fig. S3** LC-MS data of D-HPG.


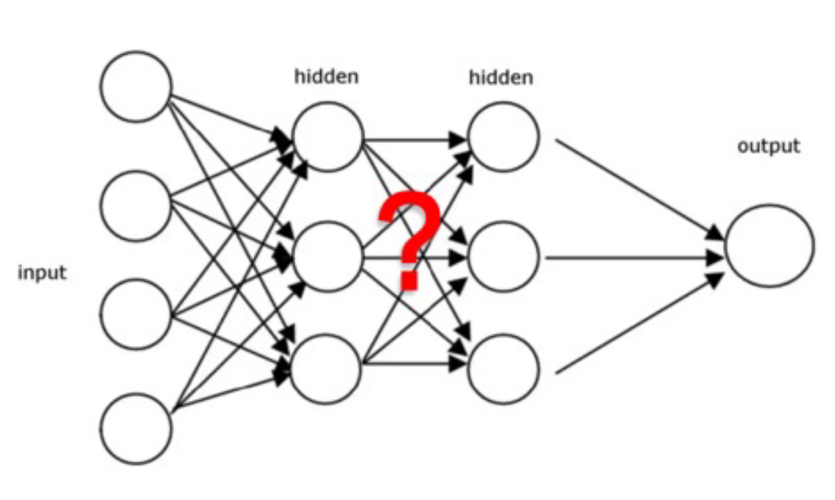


**Fig. S4** Black-box modeling from input data to generated result.


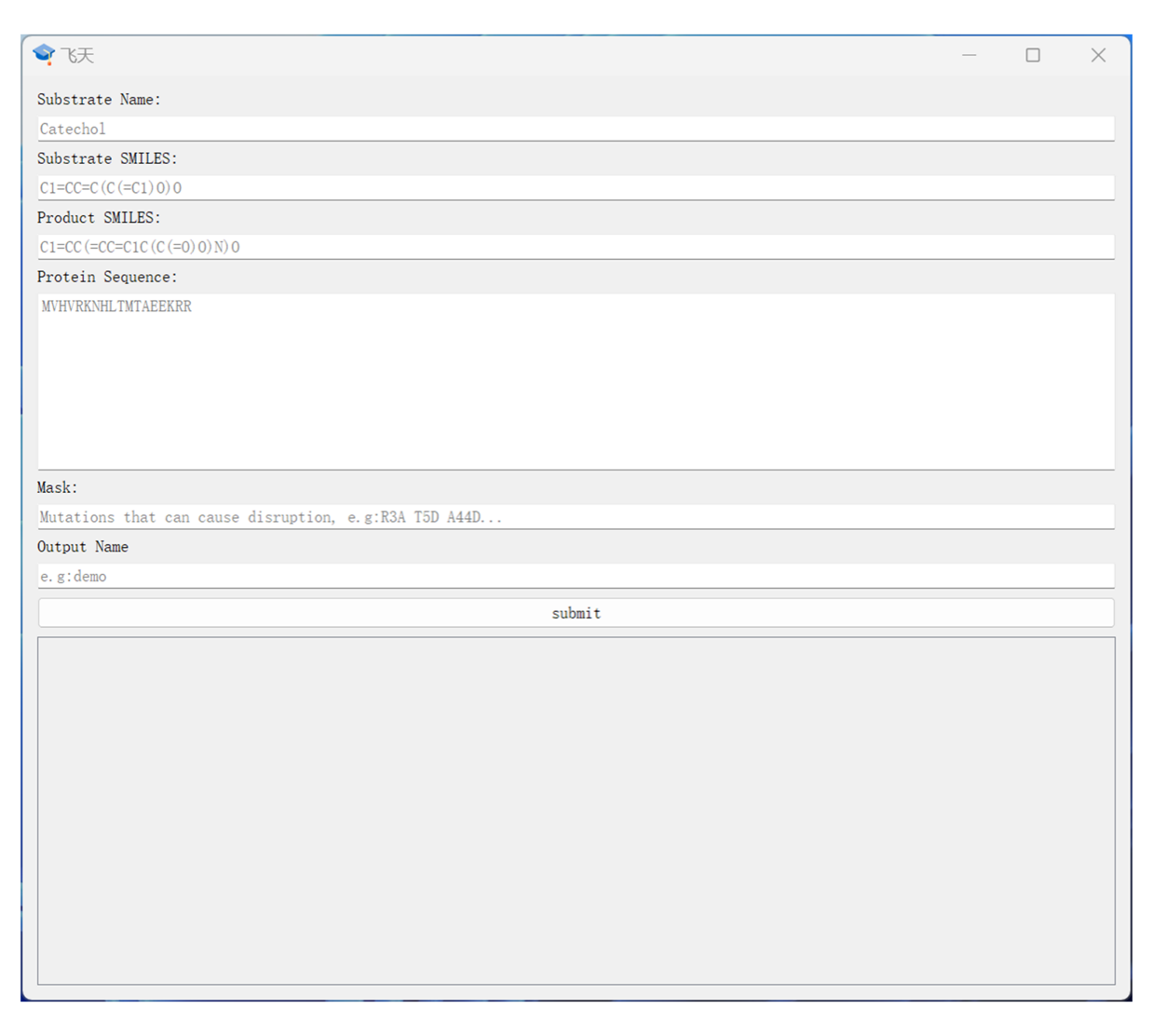


**Fig. S5** “Feitian” visualization interface.
